# A Rare Case of Non-Hodgkin B-Cell Lymphoma Following Invasive Lobular Carcinoma of the Breast: A Case Report

**DOI:** 10.3390/curroncol32040218

**Published:** 2025-04-10

**Authors:** Elisa Bertulla, Raquel Diaz, Matteo Mascherini, Marco Casaccia, Francesca Depaoli, Letizia Cuniolo, Chiara Cornacchia, Cecilia Margarino, Federica Murelli, Simonetta Franchelli, Marianna Pesce, Chiara Boccardo, Marco Gipponi, Franco De Cian, Piero Fregatti

**Affiliations:** 1 Department of Surgical and Diagnostic Integrated Sciences—DISC, University of Genova, 16132 Genova, Italy; 3500972@studenti.unige.it (R.D.); federica.murelli@unige.it (F.M.); decian@unige.it (F.D.C.); piero.fregatti@unige.it (P.F.); 2 Surgical Cinical Unit 1, Department of Surgery, IRCCS Ospedale Policlinico San Martino, 16132 Genova, Italy; matteo.mascherini@hsanmartino.it (M.M.); marco.casaccia@unige.it (M.C.); 3 Breast Surgery, IRCCS Ospedale Policlinico San Martino, 16132 Genova, Italy; francesca.depaoli@hsanmartino.it (F.D.); chiara.cornacchia@hsanmartino.it (C.C.); cecilia.margarino@hsanmartino.it (C.M.); simonetta.franchelli@hsanmartino.it (S.F.); marianna.pesce@hsanmartino.it (M.P.); chiara.boccardo@hsanmartino.it (C.B.); marco.gipponi@hsanmartino.it (M.G.)

**Keywords:** breast cancer, non-Hodgkin lymphoma, recurrence, synchronous cancer

## Abstract

The association between breast cancer and non-Hodgkin lymphoma of the spleen is extremely rare, with very few cases documented in the medical literature. We present the case of a 39-year-old woman in good health but with a family history of breast cancer, who, in 2017, developed invasive lobular carcinoma in her right breast, which was treated with mastectomy followed by hormonal therapy. In 2024, she presented with a suspicious right axillary mass, suspected of recurrence, which was confirmed by fine-needle aspiration biopsy. The patient received neoadjuvant chemotherapy, followed by axillary lymph node dissection and bilateral adnexectomy. CT and PET scans showed suspicious splenic lesions suggestive of metastases. Infectious and hematological tests were negative, leading to the decision to perform laparoscopic splenectomy. Histological examination revealed follicular B-cell non-Hodgkin lymphoma. The patient is now in good general condition and is on a biannual follow-up. The case highlights the diagnostic complexity of tumor recurrences and the need to consider alternative diagnoses other than metastasis in oncological patients.

## 1. Introduction

Breast cancer is the most common malignancy among women worldwide [1].

Advances in diagnosis and treatment have led to increased patient survival; however, this has also resulted in a higher risk of developing a second primary cancer, posing a challenge for both patients and healthcare systems [2,3].

Recent reviews estimate that individuals with a history of breast cancer have about a 25% higher risk of developing secondary tumors compared to the general population, with an even greater increase observed in those diagnosed before the age of 50 [4].

Secondary malignancies in long-term survivors may arise from sporadic cancers that would have developed regardless of environmental or genetic factors or as a consequence of breast cancer treatment [5].

Subsequent breast cancers, primarily developing in the opposite breast, are the most common [6]. It has been observed that a diagnosis of first primary breast cancer as lobular carcinoma is consistently a risk factor for developing a second primary breast cancer [7].

Among other secondary malignancies, the most frequently observed were stomach and colon cancers (27.8%), followed by endometrium and ovarian cancer (21.4%), leukemia (11.7%), lung and thyroid cancers (8.7%), melanoma (6.6%), and, least commonly, urinary tract tumors (5.5%) [8,9,10].

However, the association with lymphomas is uncommon. Breast cancer can occur as a secondary malignancy following treatment for lymphoma; in contrast, the development of lymphoma as a second malignancy after breast cancer is a rare event, with only a few reported cases [11,12].

Furthermore, in most cases of synchronous breast cancer and malignant lymphoma, the lymphoma is found either in the breast or in the ipsilateral axillary lymph nodes [13].

We present the case of a 39-year-old woman with recurrent breast cancer and a B-cell non-Hodgkin spleen lymphoma.

## 2. Case Presentation

A 39-year-old healthy woman with a family history of breast cancer underwent a right nipple-sparing mastectomy (NSM) and lymph node sampling in January 2017 after being diagnosed with a stage IIb infiltrating lobular carcinoma (ILC) (pT2 N1a). The tumor had estrogen receptor (ER) positivity of 65%, progesterone receptor (PR) positivity of 80%, Ki67 of 15%, and was negative for human epidermal growth factor receptor 2 (HER2). Metastases were detected in one out of six lymph nodes examined.

Following surgery, she was started on hormone therapy consisting of Decapeptyl (a GnRH agonist) and exemestane (an aromatase inhibitor) for a five-year regimen. During the first 30 months, Palbociclib, a CDK4/6 inhibitor, was added to her therapy to target the cell cycle and prevent cancer cell proliferation.

Genetic testing for BRCA mutations was performed and yielded a negative result. Nevertheless, in 2018, by her own choice and after psychological evaluation, the patient decided to undergo a left prophylactic mastectomy to reduce the risk of developing breast cancer in the contralateral breast. A tissue expander was initially used for breast reconstruction and subsequently replaced with a permanent implant.

Despite this aggressive treatment regimen, a follow-up CT scan of the chest and abdomen in November 2023 revealed a normal-sized spleen but with a round hypodense lesion measuring 13 × 13 mm in the middle third of the organ. Additionally, a previously known round hypodense lesion in the left adnexal region was noted.

In January 2024, a breast ultrasound detected a mixed-echo structure of 15 mm in the right axillary region, which was subsequently biopsied. The biopsy revealed the presence of recurrent infiltrating lobular carcinoma with ER of 3–5%, PR < 1%, Ki67 of 8–10%, and HER2 negativity, suggesting that the tumor had lost much of its hormone receptor expression and was becoming effectively triple-negative, a subtype of breast cancer that is generally more aggressive and harder to treat.

A subsequent PET scan showed mild tracer uptake in the splenic parenchyma at the middle third, suggesting potential metastatic involvement, but no significant tracer accumulation was noted in the right axillary nodular lesion.

From February 2024, the patient underwent 12 weeks of neoadjuvant chemotherapy consisting of carboplatin and paclitaxel, alongside scalp cooling to prevent hair loss. At the end of treatment, a follow-up breast ultrasound showed a reduction in the axillary lymphadenopathy, from 18 mm to 6 mm, suggesting a partial response to the chemotherapy.

In May 2024, a contrast-enhanced CT scan of the neck, chest, and abdomen showed that both the splenic and adnexal hypodense lesions had remained unchanged, and a new finding of a filling defect in the lumen of a small intestine loop was observed, which resembled an intussusception.

The patient then underwent a series of surgical procedures. These included right axillary lymph node dissection, which incorporated an ipsilateral lymphatic-venous anastomosis using the LYMPHA technique to improve lymphatic drainage, and bilateral adnexectomy due to a right adnexal mass and associated posterior compartment endometriosis. An exploratory laparoscopy was performed, which ultimately did not confirm the presence of intussusception.

Histological analysis of the lymph node dissection revealed carcinoma infiltrating the adipose tissue with characteristics of the lobular subtype. The recurrent tumor showed very low ER (less than 1%), PR (0%), and HER2 negativity, along with a Ki67 of 8–10%. The tumor had spread to a 15 mm area of the surrounding adipose tissue, with no recognizable lymph node structures left within the affected area. Furthermore, one lymph node in the adjacent adipose tissue contained isolated tumor cells, while the other nine lymph nodes were free of metastases.

Following the surgery, the patient was discharged on the second postoperative day in stable condition.

Given the persistence of residual disease in the axillary lymph nodes after neoadjuvant chemotherapy, the ongoing suspicion of metastatic progression, and the loss of hormone receptor expression in the recurrent tumor, capecitabine was introduced as an adjuvant therapy in June 2024, before confirming the exact nature of the splenic lesions. However, treatment with capecitabine was discontinued after the patient developed a skin rash, a common adverse effect of the drug.

In September 2024, a follow-up CT scan revealed an increase in the size of the splenic lesion (20 × 19 mm compared to 13 × 16 mm previously) and the appearance of at least four additional similar lesions in the spleen, the largest of which measured 8 × 8 mm in the upper third of the organ. These new lesions were hypodense in the arterial phase and were considered highly suspicious for secondary involvement, suggesting metastatic disease. The previously suspected intussusception in the small intestine loop was still detectable on imaging.

**Figure 1:**
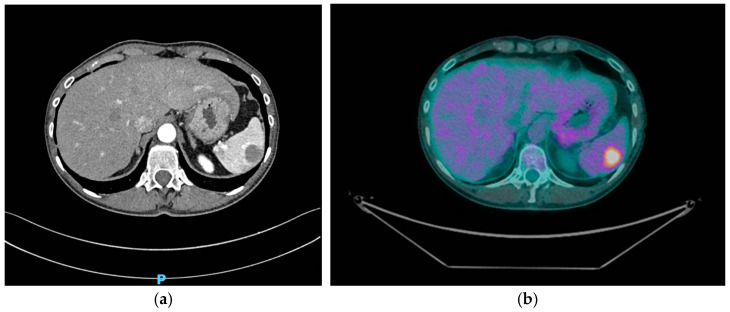
(**a**) CT scan: round hypodense spleen lesion; (**b**) PET scan: tracer uptake in the splenic parenchyma.

A subsequent PET scan confirmed an increase in both the size and metabolic activity of the focal splenic hyperaccumulation, which reinforced the suspicion of progressive disease localization in the spleen. In addition, several smaller and more defined areas of relative hyperaccumulation were observed within the spleen, indicating further spread (Figure 1).

The patient declined the proposed splenic biopsy, preferring to avoid invasive procedures at that point.

The case was then discussed in the hepatobiliary–pancreatic multidisciplinary team (MDT), where experts from oncology, surgery, and radiology collaborated to review the situation. Extensive infectious and hematologic assessments were conducted to rule out infectious causes or lymphoproliferative diseases that might have led to the splenic lesions. Tests included serology for cytomegalovirus (CMV), HIV, toxoplasmosis, Epstein-Barr virus (EBV), echinococcus, and Quantiferon, as well as two sets of blood cultures, transthoracic echocardiography, blood tests for beta-D-glucan, galactomannan, tumor markers (carcinoembryonic antigen (CEA) and cancer antigen (CA)), immunofluorescence, and the angiotensin-converting enzyme test (ACE test).

After ruling out these potential causes, the decision was to proceed with splenectomy for histopathological and microbiological analysis, as imaging alone had proven insufficient to determine the exact nature of the splenic lesions.

The patient underwent a video laparoscopic splenectomy.

Histological examination of the spleen tissue revealed the presence of a grade 1–2 non-Hodgkin follicular B-cell lymphoma (BCNHL), with expression of CD20, CD79a, CD10, Bcl-2, and Bcl-6, and absence of CD3, CD5, and Cyclin-D1. The proliferation index (Ki-67) was approximately 30–35%, indicating a moderate to high level of neoplastic cell activity.

One month after the splenectomy, the patient was in good general condition, asymptomatic, and scheduled for a six-month follow-up. This follow-up included blood tests, a breast ultrasound, and a PET scan to monitor for any further developments or recurrence of the disease.

## 3. Discussion

This case report describes the clinical course of a 39-year-old patient with right breast infiltrating lobular carcinoma (ILC), initially treated with nipple-sparing mastectomy (NSM), endocrine therapy, and adjuvant chemotherapy. Despite a multimodal therapeutic approach, the patient developed a locoregional recurrence followed by suspected splenic involvement, initially considered metastatic disease. The definitive diagnosis of classic follicular B-cell non-Hodgkin lymphoma, obtained through splenectomy, represents a rare occurrence, highlighting the need for a thorough diagnostic workup in patients with a history of breast cancer and imaging findings suggestive of recurrence.

The patient’s clinical course required a multidisciplinary approach involving oncology, surgery, radiology, and infectious disease specialists to rule out alternative causes of splenic lesions, such as infections or secondary lymphoproliferative disorders. The differential diagnosis between breast cancer metastasis and a hematologic malignancy was particularly challenging due to the absence of typical systemic lymphoma symptoms and negative blood tests for opportunistic infections. The decision to perform a splenectomy proved crucial in obtaining a definitive diagnosis and guiding further treatment.

In the literature, we found only three reported cases of breast cancer associated with splenic lymphoma (Table 1). In particular, Engin et al. [14] described splenomegaly in conjunction with a breast cancer recurrence. Additionally, the reported breast cancer type was mainly ductal carcinoma.

Our case underscores the importance of careful post-oncologic surveillance and the use of an accurate diagnostic pathway when new lesions are detected. The combination of advanced imaging and tissue biopsy is key to avoiding diagnostic delays and optimizing therapeutic management. More in-depth studies are necessary to determine the most effective treatment approaches for these patients [17].

Furthermore, the development of a hematologic malignancy in a patient with a prior history of breast cancer raises questions about the potential relationship between previous oncologic treatments and the emergence of secondary neoplasms, an aspect that warrants further investigation.

Since radiation therapy is often identified as a key cause of secondary malignancies [18], some studies, including that of Hughes et al. [19], have shown that in women over seventy who have undergone breast-conserving surgery, the addition of radiotherapy to endocrine therapy (tamoxifen) does not significantly reduce the recurrence rate or overall survival. Therefore, its omission could be considered, pending further studies.

On the other hand, as highlighted by Kang et al.’s study [20], the risk of non-Hodgkin lymphoma (NHL), particularly follicular lymphoma, is higher in young patients who have received endocrine therapy. Additionally, a study by Meattini et al. [21] comparing endocrine therapy and radiotherapy in women over the age of 70 who underwent breast-conserving surgery shows that the first one has a worse impact on quality of life than radiotherapy, despite offering a similar effect in preventing recurrences and distant metastases.

This suggests the need for further studies to explore this aspect in greater depth and reach a consistent conclusion.

## 4. Conclusions

This case highlights the importance of considering alternative causes of metastatic recurrence in patients with a history of breast cancer and atypical radiological findings. The diagnosis of B-cell non-Hodgkin lymphoma, obtained through splenectomy, underscores the need for a multidisciplinary diagnostic approach to prevent inappropriate treatments and diagnostic and therapeutic delays and to optimize clinical management.

Given the absence of standardized guidelines for surveillance in patients with a history of breast cancer and secondary hematologic malignancies, a structured follow-up approach is essential. Based on our experience, we propose a preliminary plan that could be organized as follows. However, this is not a definitive approach, and further studies are needed to refine and confirm this strategy.

1. A clinical follow-up (every 3–6 months for the first 2 years, then annually): comprehensive physical examination and blood tests, including complete blood count (CBC), lactate dehydrogenase (LDH) as a marker of lymphoma activity, tumor markers (CEA, CA 15-3), and liver and renal function tests.
2. Imaging surveillance:
  – Breast ultrasound and chest and abdominal contrast-enhanced CT scan every 6 months for the first 2 years, then annually.
  – PET-CT scan after 6 months after the last one, then every 12 months (particularly if new symptoms or imaging changes arise).

This approach ensures early detection of recurrences or secondary malignancies while minimizing unnecessary procedures. Further studies are needed to refine surveillance strategies in this complex patient population.

This case report emphasizes the value of prolonged clinical and instrumental surveillance in oncology patients and the need for further studies to explore the relationship between breast cancer and secondary hematologic malignancies. A thorough clinical evaluation, along with the integration of imaging and pathology, remains essential for early diagnosis and personalized disease management.

## Figures and Tables

**Table 1:** Review of the literature.

Sources	Age	Gender	Histology of BC	Stage	Main Findings	Surgical Treatments	Histology of NHL	Clinical Treatments
Weide, R., 1992 [15]	71	Female	Invasive ductal carcinoma	I	Hypoechogenicspleen lesions	Mastectomy, splenectomy	Low-grade NHL, centroblastic–centrocytic	NR
Ueda, Y., 2022 [16]	71	Female	Invasive carcinoma ER—PR—HER2 +	NR	Splenic mass and multiple enlarged lymph nodes	Mastectomy, axillary lymph node dissection	Diffuse large B-cell lymphoma (DLBCL)	R-CHOP therapy, radiation therapy, and trastuzumab for NHL. The patient refused chemo and radiation therapy treatment post-mastectomy.
Engin, H., 2022 [14]	66	Female	Invasive ductal carcinoma	IIA	Splenomegaly after 5 years of remission	Mastectomy, splenectomy	Marginal zone lymphoma	Chemo and radiotherapy for BC. The patient refused other treatments for NHL.

BC = breast cancer; NHL = non-Hodgkin lymphoma; ER = estrogen receptor; PR = progesterone receptor; HER 2 = human epithelial receptor-2; NR = not reported; R-CHOP = rituximab, cyclophosphamide, doxorubicin, vincristine, and prednisone.

## Data Availability

The data presented in this study are available in this article.
